# Identifying Smoking Environments From Images of Daily Life With Deep Learning

**DOI:** 10.1001/jamanetworkopen.2019.7939

**Published:** 2019-08-02

**Authors:** Matthew M. Engelhard, Jason A. Oliver, Ricardo Henao, Matt Hallyburton, Lawrence E. Carin, Cynthia Conklin, F. Joseph McClernon

**Affiliations:** 1 Department of Psychiatry and Behavioral Sciences, Duke University School of Medicine, Durham, North Carolina; 2 Department of Biostatistics and Bioinformatics, Duke University School of Medicine, Durham, North Carolina; 3 Department of Electrical and Computer Engineering, Duke University, Durham, North Carolina; 4 Department of Psychiatry, University of Pittsburgh Medical Center, Pittsburgh, Pennsylvania

## Abstract

**Importance:**

Environments associated with smoking increase a smoker’s craving to smoke and may provoke lapses during a quit attempt. Identifying smoking risk environments from images of a smoker’s daily life provides a basis for environment-based interventions.

**Objective:**

To apply a deep learning approach to the clinically relevant identification of smoking environments among settings that smokers encounter in daily life.

**Design, Setting, and Participants:**

In this cross-sectional study, 4902 images of smoking (n = 2457) and nonsmoking (n = 2445) locations were photographed by 169 smokers from Durham, North Carolina, and Pittsburgh, Pennsylvania, areas from 2010 to 2016. These images were used to develop a probabilistic classifier to predict the location type (smoking or nonsmoking location), thus relating objects and settings in daily environments to established smoking patterns. The classifier combines a deep convolutional neural network with an interpretable logistic regression model and was trained and evaluated via nested cross-validation with participant-wise partitions (ie, out-of-sample prediction). To contextualize model performance, images taken by 25 randomly selected participants were also classified by smoking cessation experts. As secondary validation, craving levels reported by participants when viewing unfamiliar environments were compared with the model’s predictions. Data analysis was performed from September 2017 to May 2018.

**Main Outcomes and Measures:**

Classifier performance (accuracy and area under the receiver operating characteristic curve [AUC]), comparison with 4 smoking cessation experts, contribution of objects and settings to smoking environment status (standardized model coefficients), and correlation with participant-reported craving.

**Results:**

Of 169 participants, 106 (62.7%) were from Durham (53 [50.0%] female; mean [SD] age, 41.4 [12.0] years) and 63 (37.3%) were from Pittsburgh (31 [51.7%] female; mean [SD] age, 35.2 [13.8] years). A total of 4902 images were available for analysis, including 3386 from Durham (mean [SD], 31.9 [1.3] images per participant) and 1516 from Pittsburgh (mean [SD], 24.1 [0.5] images per participant). Images were evenly split between the 2 classes, with 2457 smoking images (50.1%) and 2445 nonsmoking images (49.9%). The final model discriminated smoking vs nonsmoking environments with a mean (SD) AUC of 0.840 (0.024) (accuracy [SD], 76.5% [1.6%]). A model trained only with images from Durham participants effectively classified images from Pittsburgh participants (AUC, 0.757; accuracy, 69.2%), and a model trained only with images from Pittsburgh participants effectively classified images from Durham participants (AUC, 0.821; accuracy, 75.0%), suggesting good generalizability between geographic areas. Only 1 expert’s performance was a statistically significant improvement compared with the classifier (α = .05). Median self-reported craving was significantly correlated with model-predicted smoking environment status (ρ = 0.894; *P* = .003).

**Conclusions and Relevance:**

In this study, features of daily environments predicted smoking vs nonsmoking status consistently across participants. The findings suggest that a deep learning approach can identify environments associated with smoking, can predict the probability that any image of daily life represents a smoking environment, and can potentially trigger environment-based interventions. This work demonstrates a framework for predicting how daily environments may influence target behaviors or symptoms that may have broad applications in mental and physical health.

## Key Points

**Question:**

Can a deep learning approach identify environments and environmental features associated with smoking?

**Findings:**

In this cross-sectional study of 4902 images of daily environments taken by 169 smokers, a deep learning classifier was trained to identify environments associated with smoking (area under the receiver operating characteristic curve, 0.840; accuracy, 76.5%). Results generalized well across participants and geographic locations and indicated specific objects and settings associated with smoking; model predictions were significantly correlated with participant-reported craving when unfamiliar environments were viewed.

**Meaning:**

The findings suggest that a deep learning approach can be applied to trigger just-in-time adaptive cessation interventions, to optimize a smoker’s environment during a quit attempt, or to study environmental correlates of other behaviors.

## Teaser

This cross-sectional study assesses the utility of deep learning for identifying environments and objects associated with smoking.

## Introduction

Cigarette smoking results in the deaths of 500 000 US individuals a year,^1^ yet the best smoking cessation interventions, of which only a small percentage of smokers take advantage, achieve less than 20% long-term (6-month) abstinence rates.^2^ Our previous research has shown that smokers’ daily environments work against them during a quit attempt: personal smoking environments provoke craving and increase smoking behaviors even in the absence of cigarettes, lighters, and other proximal smoking cues.^3,4,5^ Indeed, viewing personal smoking environments has been linked to greater activation of a brain region associated with craving (ie, the insular cortex) compared with personal nonsmoking environments, and the degree of this activation is associated with subsequent smoking behaviors.^4^ Thus, continued exposure to specific daily environments appears to confer risk for lapse and relapse, suggesting that systematically identifying these environments and studying their characteristics might inform novel, environment-based cessation strategies.

Mobile devices and lightweight cameras now allow images and other information about daily environments to be collected on an ongoing basis. These technologies are convenient, inexpensive, and accessible to most smokers.^6^ Previous research using wearable cameras (eg, SenseCam [Microsoft Corp], HERO [GoPro], and Google Clip [Google]) has shown that a stream of everyday images can help to identify lifestyle characteristics,^7^ categorize physical activity,^8^ and detect fall risks.^9^ In addition, mobile devices can process the information that they collect in real time and interact with a user through prompts or alerts. Building on this paradigm, mobile devices make it possible to adapt an intervention to the current situation on an individual basis. This concept has been formalized as the just-in-time adaptive intervention (JITAI),^10^ which has been successfully applied to smoking cessation.^11^ The JITAI framework requires dynamic, ongoing assessment of the probability that a target behavior will occur to trigger an intervention at the most opportune moment.^12^ However, in most JITAIs, this assessment has been based on the internal state of the patient—for example, by using physiological measurements or self-report to estimate smoking risk^12,13,14,15^ or to support dieting^16^—without also considering the effects of the external environment.

Because of recent advances in deep learning, images of daily environments can now be systematically analyzed and incorporated in the JITAI framework. A type of deep learning model called the *convolutional neural network* (CNN) can be applied to identify objects and settings present in the image or make other image-related predictions. Typically, CNNs are initially trained with everyday images,^17,18^ but they have been successfully repurposed for clinical applications including identifying diabetic retinopathy^19,20^ and skin cancer.^21^ Moreover, computationally efficient CNNs (ie, with fewer parameters) have now been developed for mobile devices, allowing images to be rapidly analyzed with a smartphone or other device without substantially compromising performance.^22,23^ With a CNN-equipped smartphone or wearable device, images of daily life can be interpreted and processed in real time to predict the user’s risk of engaging in target behaviors, including smoking.

As part of a long-standing program of research on environments and smoking,^3,4,5,24,25^ 169 smokers were asked to photograph daily environments where they smoke without capturing any proximal smoking cues (eg, cigarettes or lighters) as well as daily environments where they do not smoke. The resulting images (N = 4902) were used to train a deep learning classifier to predict the probability that a given image of daily life represents a smoking environment vs nonsmoking environment. This value may then be used to determine whether new environments are likely to encourage smoking behaviors, which may be an effective proxy for smoking risk. We hypothesized that our classifier would perform better than chance and similar to smoking cessation experts, that it would generalize across participants living in 2 geographic areas (Durham, North Carolina, and Pittsburgh, Pennsylvania), and that its predictions would correlate with environment-associated craving reported by smokers. If these hypotheses were found to be valid, our model could be used to trigger a JITAI, estimate smoking patterns and craving associated with any image of daily life during a quit attempt, or retrospectively explore environmental factors that may have contributed to a relapse.

More broadly, we aimed to assess a novel framework for associating daily environments with target behaviors or symptoms. This approach could be similarly applied elsewhere in mental health (eg, mood disorders or attention-deficit/hyperactivity disorder), physical health (eg, obesogenic behaviors or allergen-induced asthma attacks), and beyond. Once the environment-behavior association is operationalized in a predictive model, environment-based interventions and therapeutic environmental modifications may be developed. Finally, given concerns about the interpretability of deep learning,^26^ we aimed to examine whether a hybrid model (deep neural network and interpretable classifier) could achieve high performance on a prediction task while still providing clinical insight.

## Methods

### Study Design and Participants

For this cross-sectional study, participants were recruited from the Durham, North Carolina (n = 106), and Pittsburgh, Pennsylvania (n = 63), areas from 2010 to 2016. Participants were active smokers (≥5 cigarettes per day for ≥1 year) aged 18 to 55 years who were ambulatory, not currently ill, and not planning to quit smoking during the study period. Those using smokeless tobacco or currently abusing alcohol or other drugs (verified with breath and urine samples) were excluded. All study procedures were approved by institutional review boards at Duke University Medical Center, Durham, or University of Pittsburgh Medical Center, Pittsburgh, and all participants signed an institutional review board–approved informed consent form before participating after receiving a complete description of the study. This study followed the Strengthening the Reporting of Observational Studies in Epidemiology (STROBE) reporting guideline.

All participants took photographs of up to 4 daily smoking environments and up to 4 daily nonsmoking environments. Smoking environments were defined as locations satisfying 2 of the following: (1) frequently visited (≥1 visit per week), (2) participant smokes 7 or more of every 10 times visited, (3) participant subjectively reports difficulty not smoking, and (4) participant rates their difficulty as 5 or more on a 10-point scale. Nonsmoking environments were defined as locations satisfying 2 of the following: (1) frequently visited (≥1 visit per week), (2) participant smokes 3 or fewer of every 10 times visited, (3) participant infrequently thinks about smoking (≤5 on a 10-point scale), and (4) participant rates their difficulty not smoking as 5 or less on a 10-point scale. For each environment, participants captured 2 images as they approached the environment and 2 from within it. Images were taken with a digital camera provided for the study, and participants were given both written and in-person camera use instructions. At a concluding study visit, a subset of Durham participants (n = 37) were shown images of 8 standard environments and asked to report the craving that they associated with each image on an 8-point scale (with 1 indicating no craving and 8 indicating extremely strong craving).

### Classification Model

**Figure 1.**
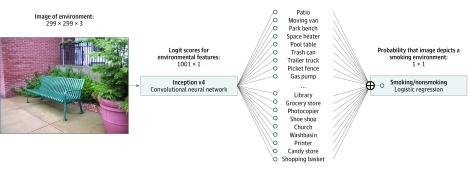
Diagram of the Final Classification Model The model extracted image features using the Inception v4 convolutional neural network and applied logistic regression to classify the images as either a smoking or nonsmoking environment.

A statistical classifier was trained to identify the images taken by study participants as either smoking or nonsmoking environments. Three distinct approaches were initially explored, as described in the eAppendix in the Supplement. The final classifier combines a pretrained image classification network—the Inception v4 CNN^27^ trained for the ImageNet large-scale visual recognition challenge^28^—with a logistic regression model trained on the current data set. This approach is consistent with the unmodified Inception v4 architecture, which uses a softmax (ie, multinomial logistic) function as its final layer,^27^ as well as the modified architectures used in other clinical applications.^21^ The first portion of the model provides information about image content in terms of 1000 categories from ImageNet. Many of these categories are common objects or locations, such as a patio, trash can, library, desk, and printer. The second portion relates information about the presence or absence of these features (ie, logit scores) to the probability that the image depicts a smoking environment vs a nonsmoking environment. Together they constitute a single model trained to classify images as smoking or nonsmoking, as illustrated in Figure 1.

The pretrained Inception v4 model was directly applied to all images. Logit values for all ImageNet large-scale visual recognition challenge categories from Inception v4 were then taken as predictors for an L2-regularized logistic regression model, in which the sum of squared model parameters is penalized to reduce overfitting. All models were implemented in TensorFlow, version 1.3.0^29^ and/or Scikit-learn, version 0.19.1^30^ machine learning frameworks for Python, version 3.5 (Python Software Foundation).

### Model Training and Evaluation

Three separate validation schemes were used to evaluate performance both within and between the 2 geographic areas (Durham and Pittsburgh). In the first validation scheme (V1), the model was initially developed and validated using images from Durham and then applied to the Pittsburgh images for secondary validation. In the second scheme (V2), this was reversed: a model initially developed and validated using the Pittsburgh images was later applied to the Durham images. In the third scheme (V3), the model was developed and validated with all images jointly.

Model training, tuning, and validation took place jointly using nested cross-validation during development.^31^ The nested cross-validation procedure avoids the biased error estimates obtained when hyperparameters are chosen to minimize outer loop error.^32^ During nested cross-validation, study participants were partitioned into 10 groups (ie, folds). For each group, a model was trained on images from participants not in that group and then evaluated on images from all participants in the group. Five folds were used in the inner loops. The regularization parameter for each outer fold was chosen as the value that maximized mean area under the receiver operating characteristic curve (AUC) over all inner folds. Cross-validation folds were defined by participant, so that all images from a given participant were assigned to the same fold. In particular, this prevented images of the same environment from being split across folds.

### Manual Classification by Experts

To contextualize classifier performance, images from 25 randomly selected participants (Durham [n = 16] and Pittsburgh [n = 9]; 732 total images) were classified by 4 smoking cessation experts (faculty and postdoctoral fellows) from the Duke University Department of Psychiatry and Behavioral Sciences who were not familiar with the current participants or data set. Experts were instructed to classify each image (yes or no) based on the following question: “Would you warn a smoker that this is an environment in which they might smoke or be tempted to smoke?” Performance was quantified in terms of sensitivity, specificity, and accuracy with respect to the image labels (smoking or nonsmoking).

### Statistical Analysis

Performances of different classifiers on the same images were compared by the DeLong test.^33^ Performances of a single classifier between cohorts (Durham or Pittsburgh) were compared by a 2-sample proportion test. Faculty member performance was compared with classifier performance by taking the sensitivity and specificity pair closest to the expert’s performance and comparing classification at that threshold with the expert’s performance by McNemar test. The number of objects detected in each cohort were compared by χ^2^ test and 2-sided Mann-Whitney *U* test evaluated at a .05 significance level.

The contribution of each ImageNet class to the model’s smoking or nonsmoking predictions was quantified using standardized logistic regression coefficients, which were placed on a common scale by dividing the nonstandardized coefficients by the SD of the corresponding predictor. To adjust for the multiple comparisons in these analyses, classifier performance comparisons were considered statistically significant only when *P* < .001.

Self-reported craving data for the 8 standard environment images were compared by Mann-Whitney *U* test, and associations between median craving and classifier predictions were compared by Spearman correlation and evaluated at a .05 significance level. Statistical analysis was performed from September 2017 to May 2018.

## Results

Of 169 participants, 106 (62.7%) were from Durham (53 [50.0%] female, mean [SD] age, 41.4 [12.0] years) and 63 (37.3%) were from Pittsburgh (31 [51.7%] female, mean [SD] age, 35.2 [13.8] years). Of 4902 images available for analysis, 3386 were from Durham (mean [SD], 31.9 [1.3] images per participant) and 1516 were from Pittsburgh (mean [SD], 24.1 [0.5] images per participant). Images were evenly split between the 2 classes, with 2457 smoking images (50.1%) and 2445 nonsmoking images (49.9%). Participant demographics are given in eTable 1 in the Supplement.

### Classifier Performance

**Figure 2.**
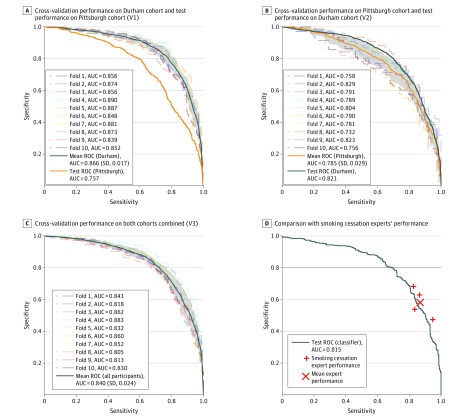
Classification Performance (Sensitivity vs Specificity Curves) A-C, Cross-validation results from the Durham cohort and subsequent test performance on the Pittsburgh cohort (A), from the Pittsburgh cohort and subsequent test performance on the Durham cohort (B), and when training using both cohorts (C). Gray shading indicates ±1 SD of the mean of results across all cross-validation folds. D, Classifier performance on a 732-image test set compared with 4 smoking cessation experts who were asked, “Would you warn a smoker that this is an environment in which they might smoke or be tempted to smoke?” AUC indicates area under the receiver operating characteristic curve; ROC, receiver operating characteristic curve.

Figure 2 shows the performance of our final model under all 3 validation schemes. The final model combines Google’s publicly available Inception v4 network^27^ with a logistic regression model; results for alternative models are presented in eTable 2 in the Supplement. Mean (SD) AUC across all cross-validation folds for the combined image set (V3) was 0.840 (0.024) (accuracy [SD], 76.5% [1.6%]). When trained on Durham images (V1), mean (SD) AUC from cross-validation was 0.866 (0.017) (accuracy [SD], 78.9% [2.3%]) compared with 0.757 (accuracy, 69.2%) when applied to the Pittsburgh images. This difference persisted when trained on Pittsburgh images (V2): mean (SD) AUC from cross-validation was 0.785 (0.029) (accuracy [SD], 72.2% [3.1%]) compared with 0.821 (accuracy, 75.0%) when applied to the Durham images. Thus, test performance was higher on the Durham images regardless of which training set was used (*P* < .001 by DeLong test). However, including the Durham images in the training set (V1 and V3) improved results compared with training with the Pittsburgh images (V2) alone (*P* < .001 by DeLong test).

### Comparison With Expert Performance

Three of 4 experts’ performance (eTable 3 in the Supplement) was above the sensitivity and specificity curve for the classifiers trained under schemes V1 to V3, as shown in Figure 2. However, these differences were statistically significant only for expert A, who outperformed the Pittsburgh-trained classifier (V2) on Pittsburgh images (χ^2^ = 6.65; *P* = .001) and the final classifier (V3) on the combined image set (χ^2^ = 4.47; *P* = .03) but not the Durham-trained classifier (V1) on Durham images (χ^2^ = 0.15; *P* = .70).

### Image-Associated Craving

**Figure 3.**
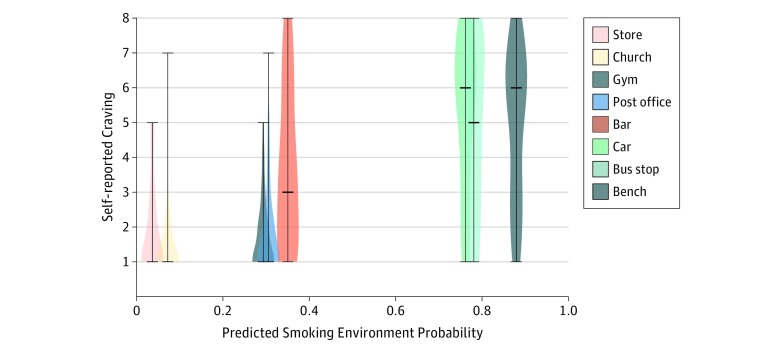
Model Predictions vs Self-reported Craving The distribution of self-reported for each of the images of typical daily environments viewed by participants. Horizontal bars indicate the median, lowest, and highest values reported. The x-axis placement indicates the probability that the image represents a smoking environment, as predicted by the model.

Classifier-predicted smoking probability for the 8 standard environments was correlated with median craving reported for that image by the study participants (ρ = 0.894; *P* = .003) (Figure 3). Predicted risk and self-reported craving were lower for all standard nonsmoking environments (store, church, gym, and post office) compared with all standard nonsmoking environments (bar, car, bus stop, and bench) (*P* < .01 for all pairwise comparisons). The classifier predicted the bar environment to be associated with lower smoking risk (35% probability) compared with the other standard smoking environments (>75% probability), and indeed, self-reported craving was lower for the bar environment compared with the other environments (bar vs car, bar vs bus stop, and bar vs bench) (*P* < .01 for all pairwise comparisons) (Figure 3).

### Classifier Interpretation

**Figure 4.**
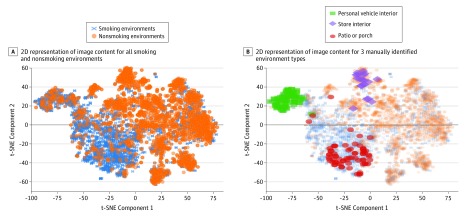
Two-Dimensional Representation of Image Content Extracted by Inception v4 A, Location of all smoking and nonsmoking images within a 2-dimensional (2D) representation of the logit layer from Inception v4 generated using t-stochastic neighbor embedding (t-SNE), a dimensionality reduction technique for high-dimensional data. B, Locations of images manually identified as personal vehicles, store interiors, and patios among the 732 manually classified images.

Examination of the logistic regression coefficients (Table) shows that patio and moving van most strongly influence the model to predict smoking, whereas library, grocery store, and photocopier most strongly influence it to predict nonsmoking. Several pieces of furniture are suggestive of nonsmoking (eg, wardrobe or desk), but pool table and entertainment center are suggestive of smoking. Figure 4 shows a 2-dimensional representation of the image content extracted by Inception v4. This representation was generated using t-stochastic neighbor embedding, a dimensionality reduction technique for visualizing high-dimensional data.^34^

**Table.** Environmental Features Ranked by Standardized Logistic Regression Coefficients^a^

Ranking	Smoking	Nonsmoking
1	Patio	Library
2	Moving van	Grocery store
3	Park bench	Photocopier
4	Space heater	Shoe shop
5	Pool table	Church
6	Trash can	Washbasin
7	Trailer truck	Printer
8	Picket fence	Candy store
9	Gas pump	Shopping basket
10	Lumber mill	Day bed
11	Cassette player	Wardrobe
12	Entertainment center	Shopping cart
13	Snake-rail fence	Bookstore
14	Mosquito net	Desk
15	Sundial	Quilt

^a^ Objects and settings detected by Inception v4 were ranked by the magnitude of their associated standardized coefficients in the final logistic regression model. Largest positive coefficients are listed as smoking, and largest negative coefficients are listed as nonsmoking.

## Discussion

This study is, to our knowledge, the first to apply deep learning to the clinically important problem of predicting whether daily environments are associated with smoking. A classifier comprising a pretrained classification network (Inception v4) coupled with a final logistic regression layer was trained and tested on a large set of smoking and nonsmoking environments photographed by smokers. The final model achieved accuracy (76.5%) significantly better than chance and comparable with human experts. Results generalized between participants and geographically distinct cohorts. When applied to unfamiliar, previously unseen environments, the model’s predictions were highly correlated with participant-reported craving associated with those environments. This provides preliminary evidence that environmental patterns associated with smoking may confer risk wherever they are encountered.

Because we chose a highly interpretable approach, this work also represents an important step toward identifying specific environmental features associated with tobacco smoking, which is the leading cause of preventable disease and death in the United States.^1^ This study should be followed by more systematic study of how daily environments promote smoking behaviors—information that can be leveraged to support more effective quitting. In clinical practice, smokers are frequently asked to identify situations and places associated with smoking (ie, triggers) and encouraged to avoid or cope with these situations when they quit. In contrast, our approach used deep learning to automatically identify objects and settings associated with smoking and can be fine-tuned to identify an individual smoker’s specific environmental triggers given adequate initial training. The model differentiated between public settings likely to be associated with smoking (eg, park bench and gas pump) vs public settings not likely to be associated with smoking (eg, grocery store and church) and between home settings likely to be associated with smoking (eg, patio and entertainment center) vs home settings not likely to be associated with smoking (eg, washbasin and wardrobe). Of importance, however, the model considers all objects and settings jointly when making its predictions.

Whereas in the current research, environments associated with smoking were predicted using photographs taken by smokers and brought into the laboratory, we hypothesize that a similar approach can be applied to predict smoking risk in real time. Just-in-time adaptive interventions^10,16^ depend on quick and accurate prediction of risk (ie, just-in-time) and information about the nature of the risk to deliver situationally relevant (ie, adaptive) interventions. We envision a JITAI wherein images from a personal, wearable camera or smart glasses are assessed on an ongoing basis to quantify smoking risk and trigger an intervention when risk is high. By choosing a high-sensitivity operating point (eg, 90% sensitivity and 50% specificity) (Figure 2), the current model could be tested as a means to trigger JITAIs supporting smoking cessation. Additional research is needed to further improve classification performance, translate prediction models into clinical interventions, and explore how smoking environments relate to prospective smoking risk. Our approach does not yet distinguish between environments where smoking takes place and those that increase urge, which may be critical to the success of an environment-based intervention. Subsequent work could explore this distinction, for example, through ecological momentary assessment of craving or by analyzing a sequence of images taken in the moments leading up to a smoking event.

Connecting the external environment to smoking risk also informs a range of environment-based interventions, in which information about prosmoking environments can be used during a quit attempt. For example, images of a potential destination (eg, acquired from a website) could be analyzed before visiting to estimate whether that environment might increase craving. In this way, our approach could be used to preempt lapse triggers in addition to identifying them in real time. Alternatively, the model could support therapeutic environmental modifications by which features promoting healthy behaviors are incorporated and features contributing to unhealthy behaviors are systematically identified and altered. For example, images collected during a failed quit attempt could be analyzed to pinpoint environmental factors associated with lapse or relapse. Working together with a clinician, the smoker might then remove these factors from their personal environments before their next quit attempt to increase the chance of success.

To support these applications, it is important that all prediction was across participants (ie, out-of-sample prediction). This was ensured by the participant-wise partitions used in our nested cross-validation procedure. That is, predictions for a given participant were made by a model trained only on other participants. The model also generalized well between geographically distinct cohorts (Durham and Pittsburgh) and accurately predicted the level of craving the participants associated with locations that they had not previously seen. This suggests that the model may be capable of predicting smoking risk associated with a range of familiar and unfamiliar environments encountered in daily life. Importantly, it relies only on features in the surrounding environment and not on proximal smoking cues, which might provide information about smoking not related to the environment itself.

Although our results generalized across participants, personalization might further improve model performance by identifying objects and settings associated with smoking on an individual basis. A personalized approach would ensure that performance is consistent across distinct groups of smokers, such as those who smoke in the home vs those who do not. We intend to explore the potential benefit of personalization in future work. If beneficial, our model can easily be personalized by fine-tuning the measures of its final layer, which directly associates objects and settings detected in daily environments with smoking environment status. Other technical strengths of this work include our exploration of multiple deep learning approaches (eTable 3 in the Supplement) and the robustness of our results when varying the final classification layer (eg, linear discriminant analysis and multilayer perceptron).

### Limitations

This work has several important limitations. First, participants in the parent studies were instructed to photograph locations where smoking was highly likely and highly unlikely to take place. A larger, more naturalistic image set is needed to show that accurate prediction extends to the full range of environments encountered in smokers’ daily lives. Second, some objects and settings (eg, personal vehicles) were smoking environments for some participants and nonsmoking environments for others (Figure 3), which may suggest that there are more subtle differences between these environments that our architecture was not able to identify. Alternatively, this finding may indicate a need for personalized models, which would require a larger data set with more comprehensive coverage of each participant’s daily environments. Third, our prediction model was limited by the objects and settings identified by Inception v4 and did not account for interactions between environmental features or higher-order features (eg, inside or outside) that may be important determinants of smoking status. In subsequent work, it may be important to revisit the effectiveness of alternative, hierarchical classification models if trained using a larger data set. Fourth, smokers were instructed to remove proximal smoking cues (eg, lighters and cigarettes) before photographing their environment to avoid cue reactivity in the parent study. As previously discussed, this allowed us to isolate the association between the external environment and smoking risk. However, retraining on image sets that include these objects may allow us to improve performance for the purpose of triggering a JITAI. Fifth, smokers in these studies were adult, daily, non–treatment-seeking smokers who smoked 5 or more cigarettes per day. Additional work is needed to evaluate whether classification accuracy extends to other segments of the smoker population, with an emphasis on treatment-seeking smokers, and to assess the feasibility and acceptability of monitoring personal daily environments.

## Conclusions

The findings suggest that objects and settings found in images of daily life can be used to identify environments associated with smoking, which may in turn be an effective proxy for craving and smoking risk. Environment-based risk predictions generalized between participants and geographic locations, suggesting that specific environmental patterns are consistently associated with smoking. A deep learning approach can be used to (1) identify environmental features associated with and antecedent to smoking behavior, (2) predict smoking or nonsmoking status associated with any image of daily life, and (3) trigger just-in-time, adaptive, environment-based cessation interventions. Each of these directions could be coupled with existing self-monitoring interventions to improve our ability to help smokers quit, in turn reducing disease and death from smoking. More broadly, this work showed a framework for interpreting and predicting the influence of daily environments on other target behaviors or symptoms—one with numerous applications in mental health (eg, mood disorders and attention-deficit/hyperactivity disorder), physical health (eg, obesogenic behaviors and allergen-induced asthma attacks), and beyond. Understanding how the external environment affects behaviors or symptoms of interest may inform environment-based interventions and therapeutic environmental modifications.

## Notes

Supplement. **eAppendix.** Comparison of All Classifiers and Cohort Demographics **eTable 1.** Demographics of Durham, NC and Pittsburgh, PA Participants **eTable 2.** Detailed Classifier Performance **eTable 3.** Expert Classification Details **eReferences**
