# Supplementary material for: Identifying Smoking Environments From Images of Daily Life With Deep Learning
Source: JAMA Netw Open. 2019 Aug 2;2(8):e197939. doi: 10.1001/jamanetworkopen.2019.7939 (PMC6681554; doi:10.1001/jamanetworkopen.2019.7939)
Supplement: Supplement. — eAppendix. Comparison of All Classifiers and Cohort Demographics eTable 1. Demographics of Durham, NC and Pittsburgh, PA Participants eTable 2. Detailed Classifier Performance eTable 3. Expert Classification Details eReferences [file jamanetwopen-2-e197939-s001.pdf]

Supplementary Online Content

Engelhard MM, Oliver JA, Henao R, et al. Identify smoking environments from images of daily life with deep learning. *JAMA Netw Open*. 2019;2(8):e197939. doi:10.1001/jamanetworkopen.2019.7939

eAppendix. Comparison of All Classifiers and Cohort Demographics

eTable 1. Demographics of Durham, NC and Pittsburgh, PA Participants

eTable 2. Detailed Classifier Performance

eTable 3. Expert Classification Details

eReferences

This supplementary material has been provided by the authors to give readers additional information about their work.

© 2019 Engelhard MM et al. *JAMA Network Open.*

eAppendix: Comparison of All Classifiers and Cohort Demographics

Three approaches to classification comprising several specific classifiers were initially explored. Two were based on the Inception v4 CNN previously described (Approaches 1 and 2), and the third utilized a Faster-RCNN object detection network[1] based on the ResNet CNN architecture[2] and pre-trained on the Common Objects in Context (COCO) dataset (Approach 3).[3] All classifiers were trained and evaluated using nested cross-validation,[4] and the same CV partitions were used for each classifier. Numeric and categorical hyperparameters were selected as the median or mode, respectively, of the optimal values found in each inner loop. The final model was selected due to its competitive performance (i.e. no statistically significant differences compared to other Approach 1 models) and the interpretability and familiarity of logistic regression.

The final classifier (Inception v4 + L2-regularized Logistic Regression) had highest AUC and accuracy under all three validation schemes (i.e. trained via cross-validation with Durham images, trained via cross-validation with Pittsburgh images, trained via cross-validation with all images). However, several other Approach 1 models had similar performance; for example, a second Approach 1 model (Inception v4 + MLP) model had similar AUC when trained on Duke and combined image sets (0.855 and 0.828, respectively), and a third Approach 1 model (Inception v4 + LDA) had similar accuracy (78.6% and 76.3%, respectively). Detailed performance for all classifiers (mean ± SD of AUC and accuracy across all CV folds for all image sets) may be found in eTable 3.

Differences in AUC between classifiers of the same approach were not statistically significant ($p>10^{-4}$). In contrast, differences in AUC between approaches were statistically significant ($p<10^{-4}$): Approach 1 performed better than Approach 2, which in turn performed better than Approach 3. The one exception was the Pittsburgh image set, where differences between Approaches 1 and 2 were not statistically significant ($p>10^{-4}$).

Description of Approach 1-3 Classifiers

Approach 1: Inception v4 + Classifier

These classifiers follow the approach described in the main text, in which the output logits from the pre-trained Inception v4 model were used as predictors to train a smoking/nonsmoking classifier in Scikit-learn 0.19.1.[5] In addition to L2-regularized logistic regression, we explored: (1) L1-regularized logistic regression, (2) a multi-layer perceptron (MLP) with a single hidden layer, and (3) linear discriminant analysis. Hyperparameters tuned by nested CV included regularization parameters and the number of MLP hidden units.

Approach 2: Inception v4 Retraining

The Inception v4 network was modified and fine-tuned to directly classify images as smoking/nonsmoking. Specifically, the final two layers (logit and softmax) were modified for our two-class problem and randomly initialized. The network was then trained in Tensorflow via stochastic gradient descent (ADAM optimizer[6], learning rate = 10^{-4}, dropout $p_{keep} = 0.8$) with mini-batches of 60 images to minimize average cross-entropy over the training set for each outer fold. The number of training epochs was chosen by nested CV: training proceeded until average cross-entropy over the inner fold validation set exceeded 105% of its minimum.[7]

Approach 3: Faster-RCNN-ResNet + Classifier

A COCO-trained Faster-RCNN-ResNet model was directly applied to all images via Tensorflow to detect objects included in the 90 COCO object classes. Object class counts were then taken as predictors for a classification model trained on the current dataset. Five classifiers were explored: (1) L1- and (2) L2-regularized logistic regression, (3) multi-layer perceptron with a single hidden layer, (4) Bernoulli naïve Bayes, and (5) multinomial naïve Bayes. These classifiers were implemented in Python 3.5 via Scikit-learn 0.19.1.

Objects per Image

The number of objects detected per image (via Faster-RCNN-ResNet) was higher for the Durham images ($p=0.004$), with a greater proportion of images having ≥ 2 objects (77.7% Durham, 68.5% Pittsburgh; $p<0.001$).

© 2019 Engelhard MM et al. *JAMA Network Open.*

eTable 1: Demographics of Durham, NC and Pittsburgh, PA participants.

	All	Durham	Pittsburgh	p-value
**N**	169	106	63	
**Age**, mean±SD [range]	39.1±13.0 [18 – 65]	41.4±12.0 [19 – 65]	35.2±13.8 [18 – 63]	0.003
**Sex**, F:M, (%F)	84:82 (50.6%)	53:53 (50.0%)	31:29 (51.7%)	0.96
**Race**, N (%)				<0.001
White	87 (52.4%)	43 (40.6%)	44 (73.3%)	
American Indian	0 (0.0%)	0 (0.0%)	0 (0.0%)	
Asian	7 (4.2%)	4 (3.8%)	3 (5.0%)	
Black	68 (41.0%)	58 (54.7%)	10 (16.7%)	
Native Hawaiian / Pacific Islander	0 (0.0%)	0 (0.0%)	0 (0.0%)	
More than one	4 (2.4%)	1 (0.9%)	3 (5.0%)	
Unknown / other	0 (0.0%)	0 (0.0%)	0 (0.0%)	
**Ethnicity**, N (%)				0.37
Non-Hispanic	161 (97.0%)	103 (97.2%)	58 (96.7%)	
Hispanic	1 (0.6%)	0 (0.0%)	1 (1.7%)	
Unknown	4 (2.4%)	3 (2.8%)	1 (1.7%)	
**FTND Total**, median [IQR] [range]	5 (3 – 6) [0 – 10]	5 (3 – 6) [0 – 10]	4 (3 – 6) [0 – 10]	0.80
**Cigarettes per day**, mean±SD [range]	15.3±6.3 [4 – 40]	14.8±6.6 [4 – 40]	16.1±5.6 [10 – 40]	0.07

*Demographics not available for 3 Pittsburgh participants

SD: standard deviation; IQR: interquartile range; FTND: Fagerstrom Test for Nicotine Dependence

eTable 2: Detailed Classifier Performance

		Durham				Pittsburgh				Both			
**Approach**	**Details**	**Avg Acc**	**STD Acc**	**Mean AUC**	**STD AUC**	**Avg Acc**	**STD Acc**	**Mean AUC**	**STD AUC**	**Avg Acc**	**STD Acc**	**Mean AUC**	**STD AUC**
Inception v4 pre-trained + classifier	MLP -- 1 hidden layer	77.7%	5.3%	0.855	0.043	68.7%	3.1%	0.752	0.047	75.0%	2.4%	0.828	0.024
	Logistic Regression -- L2 Reg	78.9%	2.3%	0.866	0.017	72.2%	3.1%	0.785	0.029	76.5%	1.6%	0.840	0.024
	Logistic Regression -- L1 Reg	77.9%	5.2%	0.846	0.049	69.1%	5.4%	0.754	0.064	74.9%	3.0%	0.824	0.027
	LDA	78.6%	5.0%	0.849	0.05	68.7%	4.2%	0.748	0.056	76.3%	3.3%	0.826	0.03
Inception v4 fine-tuned	SGD, ADAM Optimizer	75.9%	5.5%	0.826	0.058	67.2%	6.3%	0.733	0.080	72.7%	3.4%	0.798	0.032
Faster-RCNN ResNet COCO pre-trained + classifier	Bernoulli Naïve Bayes	68.7%	5.3%	0.742	0.064	59.7%	6.5%	0.61	0.085	67.0%	2.4%	0.702	0.031
	Multinomial Naïve Bayes	65.6%	4.7%	0.742	0.061	59.5%	7.7%	0.623	0.088	63.8%	3.0%	0.707	0.033
	Logistic Regression -- L2 Reg	69.6%	4.6%	0.752	0.059	58.2%	6.8%	0.62	0.087	66.3%	2.8%	0.713	0.033
	Logistic Regression -- L1 Reg	68.8%	4.7%	0.751	0.059	55.5%	5.3%	0.62	0.081	65.6%	2.6%	0.709	0.035
	MLP -- 1 hidden layer	69.3%	5.8%	0.747	0.063	57.1%	4.6%	0.622	0.074	66.3%	2.8%	0.709	0.036

Performance statistics are broken down by approach (Inception v4 + Classifier, Inception v4 Retraining, or Faster-RCNN-ResNet + Classifier), final-layer classifier (e.g. logistic regression, multi-layer perceptron), and training cohort(s) (Durham, Pittsburgh, or both).

© 2019 Engelhard MM et al. *JAMA Network Open.*

eTable 3: Expert Classification Details

Image Set	Smoking Cessation Expert	True Pos	True Neg	False Pos	False Neg	Sens	Spec	Acc
**Durham**	A	243	120	140	13	0.949	0.462	0.703
	B	218	140	120	38	0.852	0.538	0.694
	C	221	165	95	35	0.863	0.635	0.748
	D	223	178	82	33	0.871	0.685	0.778
	Average	226.25	150.75	109.25	29.75	0.884	0.580	0.731
**Pittsburgh**	A	102	54	54	6	0.944	0.500	0.722
	B	86	58	50	22	0.796	0.537	0.667
	C	94	66	42	14	0.870	0.611	0.741
	D	78	73	35	30	0.722	0.676	0.699
	Average	90	62.75	45.25	18	0.833	0.581	0.707
**Combined**	A	345	174	194	19	0.948	0.473	0.709
	B	304	198	170	60	0.835	0.538	0.686
	C	315	231	137	49	0.865	0.628	0.746
	D	301	251	117	63	0.827	0.682	0.754
	Average	316.25	213.5	154.5	47.75	0.869	0.580	0.724

Results of classification of a random Sample of 732 Images (516 Durham, 216 Pittsburgh) by smoking cessation experts

© 2019 Engelhard MM et al. *JAMA Network Open.*

eReferences

1. Ren S, He K, Girshick R, Sun J. Faster R-CNN: Towards Real-Time Object Detection with Region Proposal Networks. *IEEE Trans Pattern Anal Mach Intell*. 2015;39(6):1137-1149. doi:10.1109/TPAMI.2016.2577031.

2. He K, Zhang X, Ren S, Sun J. Deep Residual Learning for Image Recognition. In: *2016 IEEE Conference on Computer Vision and Pattern Recognition (CVPR)*. IEEE; 2016:770-778. doi:10.1109/CVPR.2016.90.

3. Lin T-Y, Maire M, Belongie S, et al. Microsoft COCO: Common Objects in Context. In: Springer, Cham; 2014:740-755. doi:10.1007/978-3-319-10602-1_48.

4. Varma S, Simon R. Bias in error estimation when using cross-validation for model selection. *BMC Bioinformatics*. 2006;7(1):91. doi:10.1186/1471-2105-7-91.

5. Pedregosa F, Varoquaux G, Gramfort A, et al. Scikit-learn: Machine Learning in Python. *J Mach Learn Res*. 2011;12(Oct):2825-2830. http://jmlr.csail.mit.edu/papers/v12/pedregosa11a.html. Accessed February 5, 2018.

6. Kingma DP, Ba J. Adam: A Method for Stochastic Optimization. December 2014. http://arxiv.org/abs/1412.6980. Accessed February 6, 2018.

7. Prechelt L. Automatic early stopping using cross validation: Quantifying the criteria. *Neural Networks*. 1998;11(4):761-767. doi:10.1016/S0893-6080(98)00010-0.

© 2019 Engelhard MM et al. *JAMA Network Open.*
